# Development of a comprehensive measure of spatial access to HIV provider services, with application to Atlanta, Georgia

**DOI:** 10.1186/s40064-016-2515-8

**Published:** 2016-07-04

**Authors:** Sharoda Dasgupta, Michael R. Kramer, Eli S. Rosenberg, Travis H. Sanchez, Patrick S. Sullivan

**Affiliations:** Laney Graduate School, Emory University, Mailstop 1000-001-1AF, 209 Administration Building, 201 Dowman Drive, Atlanta, GA 30322 USA; Department of Epidemiology, Rollins School of Public Health, Emory University, 1518 Clifton Road NE, Atlanta, GA 30329 USA

**Keywords:** HIV, Spatial access, Healthcare services, Travel mode

## Abstract

**Background:**

No existing measures of HIV care access consider both spatial proximity to services and provider-related characteristics in a single measure. We developed and applied a tool to: (1) quantify spatial access to HIV care services (supply) and (2) identify underserved areas with respect to HIV cases (demand), by travel mode, in Atlanta.

**Methods:**

Building on a study of HIV care engagement, data from an HIV care provider database, and HIV case counts by zip code tabulation area (ZCTA) from AIDSVu.org, we fit a discrete choice model to estimate practice characteristics most salient in defining patient care access. Modified spatial gravity modeling quantified supply access based on discrete choice model results separately for travel by car and by public transportation. Relative access scores were calculated by ZCTA, and underserved areas (defined as having low supply access and high HIV case count) were identified for each travel mode.

**Results:**

Characteristics retained in the final model included: travel distance, available provider-hours, availability of ancillary services, and whether Ryan White patients were accepted. HIV provider supply was higher in urban versus suburban/rural areas for both travel modes, with lower supply access if traveling by public transportation. Underserved areas were concentrated in south and east Atlanta if traveling by public transportation, overlapping with many areas of high poverty. Approximately 7.7 %, if traveling by car, and 64.3 %, if traveling by public transportation, of Atlanta-based persons with diagnosed HIV infection resided in underserved areas.

**Conclusion:**

These findings highlight underserved areas in south and east Atlanta if traveling by public transit. Conceptualizing access to medical services spatially and by travel mode may help bridge gaps between patient needs and service availability and improve HIV outcomes.

**Electronic supplementary material:**

The online version of this article (doi:10.1186/s40064-016-2515-8) contains supplementary material, which is available to authorized users.

## Background

Clients of the healthcare system choose providers based on personal needs or constraints, such as travel distance, health insurance, and convenient hours to receive services. Access to medical care refers to the fit or match between the supply of available healthcare services and the demand for services based on patient needs, and is characterized by the use of healthcare services (Penchansky and Thomas 1981; Andersen 1995). Access to care is a function of many components, including local availability of medical providers as well as the attributes of these providers, and thus, could be conceptualized as being inherently spatial in nature. For the remainder of this paper, we refer to access to care as “spatial access to care.”

Identifying mismatches between supply and demand for medical services is key to addressing gaps in HIV care engagement (Sagrestano et al. 2013; Conover and Whetten-Goldstein 2002; Reif et al. 2005) and other clinical outcomes (Robbins et al. 2007; Mugavero et al. 2013; Institute of Medicine Committee 1993) in Atlanta, where case burden is high (Centers for Disease Control and Prevention 2015; HIV Surveillance Fact Sheet 2013) and HIV prevalence varies geographically across the metropolitan area (Hixson et al. 2011). However, spatial access to healthcare services is a difficult construct to quantify because of at least two reasons: (1) there is no standard model of conceptualization, and (2) it is difficult to find data which incorporate multiple dimensions of access.

Based on previous frameworks, we conceptualized spatial access as encompassing the following domains: availability and accessibility of providers (characterized by density of available providers and measures of spatial proximity, such as travel distance, commute time, and travel mode), affordability of services, acceptability of services based on patient-provider interactions, and accommodation based on hours of operation (Penchansky and Thomas 1981). Building on previous work on spatial access (Bell et al. 2013; Luo and Wang 2003; Guagliardo 2004; Crooks and Schuurman 2012; Rosero-Bixby 2004), we comprehensively quantified spatial access to care by incorporating multiple facets of access, including availability, accessibility, affordability, and accommodation, into a single measure. We also conceptualized spatial access to services separately for travel by car and by public transportation because we hypothesized, a priori, that (1) longer commute times when traveling by public transportation are associated with reduced accessibility of services, compared with traveling by private transportation (Dasgupta et al. 2015a), and (2) characteristics related to patients selecting an HIV provider may vary based on social determinants of health correlated with public transportation use, such as poverty (Glaeser et al. 2008; Dasgupta et al. 2015b).

In this study, we used discrete choice modeling and modified spatial gravity modeling to develop a novel tool to (1) comprehensively quantify spatial access to services (supply), and (2) identify underserved areas, with respect to HIV case burden (demand), by mode of transportation in the Atlanta area.

## Methods

**Fig. 1:**
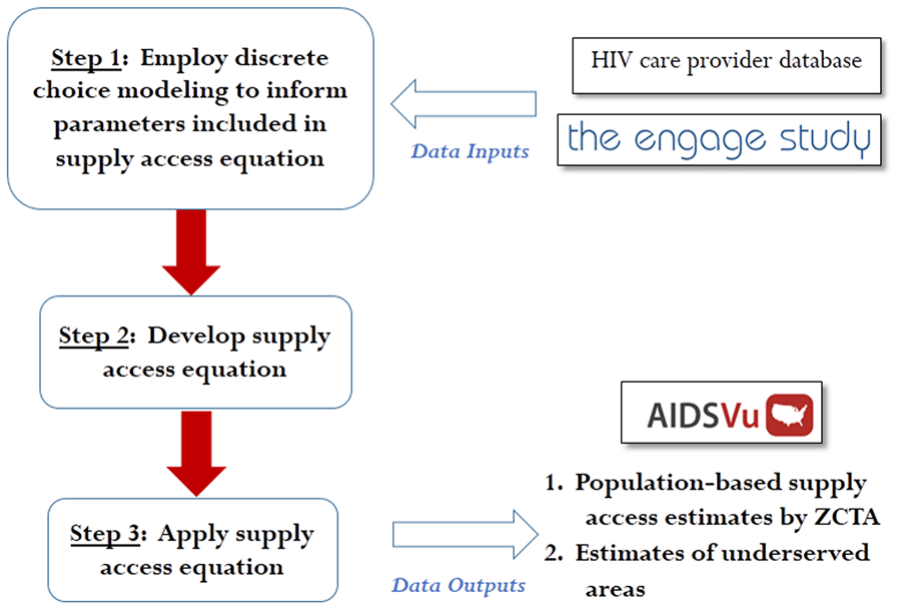
A schematic of the three-step process employed to quantify supply access to services and identify underserved areas in Atlanta

As an overview, we employed a multi-step process and three different data sources to address the study objectives. In the first step, discrete choice modeling informed the parameters included in a supply access equation. Second, we constructed the supply access equation, which described spatial access to the supply of Atlanta HIV providers by zip code tabulation area (ZCTA) as a function of factors determined by results from the discrete choice model. Finally, the supply access equation was applied to quantify two study outputs: (1) population-based estimates of supply access by ZCTA and (2) estimates of underserved communities in the Atlanta area. For this study, Atlanta was defined by the six county area, which included Clayton, Cobb, Dekalb, Douglas, Fulton, and Gwinnett counties. A schematic of this three step process, along with the data sources used in each step, is highlighted in Fig. 1. Information on the analytic methodology employed in this analysis are outlined in subsequent sections, and more details on model assumptions are discussed in Additional file 1: Appendix S1.

### Data sources

#### HIV care provider database

We created a database of key characteristics about each of the major clinics or practices located in the six county Atlanta area, identified through multiple sources of data (including the Southeast AIDS Training and Education Center Key Contacts book, the Ryan White medical provider directory available through the HRSA data warehouse, the AIDS.gov resource directory, and the Georgia Care and Prevention in the United States (CAPUS) resource hub). We called every practice or clinic to verify that HIV primary care was provided on-site by at least one physician, physician’s assistant, or nurse practitioner. After excluding locations that did not meet this definition, 41 HIV clinics and practices remained. Information on facility type, accepted payment options, availability of ancillary services, and the number of available providers and weekly appointment hours was collected from each practice or clinic.

We classified each facility as a private practice, clinical research facility, community health center or community-based service organization, or state or local health department. To assess patient eligibility based on payment options, we asked each clinic whether it accepted private health insurance, Medicare, or Medicaid as forms of payment, offered a discounted pay structure for those who qualified based on income, and provided Ryan White services to patients. First established in 1990, the Ryan White HIV/AIDS program is a federal grant system which works with local providers to cover the cost of medical care and support services for persons living with HIV who would otherwise be unable to afford such services. Data were also collected on whether or not clinics offered the following ancillary services: HIV case management, mental health services, dental care, substance abuse treatment, transportation assistance, and an on-site pharmacy that fills prescriptions. The number of part-time and full-time providers and weekly appointment hours were quantified and used to estimate the number of provider-hours available to clients per week. We computed descriptive statistics on the availability of weekly provider-hours, accepted payment types, and availability of ancillary services overall, and by provider type. We evaluated differences in these characteristics by provider type using a Mantel-Haenszel chi-square test for categorical variables and a Wilcoxon-Mann-Whitney test for continuous variables.

#### The Engage Study

The Engage Study investigated structural and psychosocial barriers to HIV care among self-identifying HIV-positive men who have sex with men (MSM) living in the Atlanta area. Details on recruitment have been previously described (Dasgupta et al. 2014). All participants completed an online questionnaire that collected data on residential address at the time of the interview, the location of the last attended HIV care provider, and primary modes of travel taken to attend appointments. We used this information to estimate travel distance and commute time to attend HIV care visits based on reported mode of travel (by car versus public transit) using the Google Maps Direction application program interface (API) (Dasgupta et al. 2014, 2015b). Latitude-longitude coordinates for residence were anonymized before being entered into Google maps to protect confidentiality of participants. Only participants who were living in the six county Atlanta area and reported receiving HIV care from a provider in the six county area in the previous year were included in the analysis. Descriptive statistics on the sample included in the analysis are described.

#### AIDSVu

We obtained HIV case counts by ZCTA in the six county Atlanta area from AIDSVu.org, an online mapping tool which illustrates rates of HIV for several cities across the United States. Data reflected the number of prevalent cases reported through the end of 2011. Only data for ZCTAs whose population-weighted centroids were contained in the six county area were included in this study.

### Discrete choice modeling

The Engage Study data were used in a discrete choice model to estimate characteristics most important in selecting a provider, by mode of transportation taken to attend HIV care visits. Characteristics spanning four of the dimensions of spatial access (availability, accessibility, affordability, and accommodation) were considered in describing the supply environment, and thus, were contributors to the individual’s choice utility function. Facility-specific characteristics included: provider type (private practice versus other), whether or not any ancillary services were offered (e.g., transportation assistance, substance abuse treatment), payment options (whether or not Ryan White patients were accepted), the number of available provider-hours during the week, and whether or not walk-in hours were offered. Two covariates directly related to the participant were also considered as potential descriptors of supply access, including travel distance between study participant residence and each HIV provider and the mode of transportation taken to attend HIV care visits. Because travel distance and provider-hours were non-normally distributed, the natural-log of these two variables were included in the discrete choice modeling and evaluation of supply access.

We employed a generalized estimating equations (GEE) model with a logit link function to investigate factors associated with choosing an HIV care provider. We accounted for clustering by patient using an exchangeable correlation structure. Because assessing potential differences in choice of HIV care provider by travel mode was of primary interest, we included two-way interaction terms between each provider characteristic and mode of transportation taken to attend HIV visits. Backward selection was used to determine which variables should be retained in the final model, using a cutoff of p < 0.05. The multivariable GEE model was built using SAS 9.4 (Cary, NC).

### Development of supply access equation

Spatial gravity modeling assumes an exponential relationship between travel distance and access to care, such that increasing travel distance results in decreased access (Crooks and Schuurman 2012). The supply access equation represented a modified gravity model that generalized spatial access to HIV care across the entire Atlanta study area based on regression coefficients from the discrete choice model. These coefficients were used as corresponding weights of importance for each characteristic included in the equation. A single score generated from the equation represented supply access from the population-weighted centroid of a given ZCTA to an individual HIV provider, given the sum of individual provider-related characteristics, road distance between the centroid and provider, and the mode of transit.

### Application of supply access equation

#### Study output 1: computing population-based estimates of supply access by ZCTA

For every ZCTA, 41 supply access scores were generated for travel by public transit, and 41 supply access scores were generated for travel by car. For each mode of transit, these individual supply access scores for ZCTA-provider pairs were summed by ZCTA, presenting a single estimate of the average supply access to HIV care providers available for a given ZCTA. Summary scores were computed separately for travel by car and travel by public transportation. Based on previously published results, we also transformed supply access scores to account for the barrier to HIV care attendance associated with traveling by public transit, versus by car, among people living with HIV in Atlanta (Dasgupta et al. 2015b). The differences in supply access scores between two modes of travel were highly sensitive to the estimate we used to transform scores. Thus, we also present results from a sensitivity analysis which explores potential consequences of using different values to transform spatial access scores in Additional file 2: Figure S1.

Using ArcGIS 10.2, supply access scores were mapped for travel by car and by public transportation. The geographic distribution of scores were compared across travel mode using quintiles of supply access for travel by car. Differences in scores across urban versus suburban and rural areas for each travel mode were also assessed. Urban areas were defined as areas inside the main auxiliary highway, or loop route, of Atlanta, while suburban and rural areas represented neighborhoods outside this highway.

#### Study output 2: identifying underserved areas

After evaluating supply access to HIV care providers by ZCTA, potentially underserved areas in the six county area were identified by mode of transit. We defined underserved areas as ZCTAs with overlapping areas of low supply access (one of the two lowest quintiles) and high HIV case count (one of the two highest quintiles). Subsequently, we estimated the proportion of all HIV cases in Atlanta living in these underserved areas for each mode of travel. Population-based estimates of poverty by ZCTA (areas in which >20 % of the population are living in poverty) were obtained from the American Community Survey (five year estimates, 2009–2013) to quantify potential overlap with underserved areas.

## Results

### Study population

#### HIV care provider database

The average number of weekly provider-hours was lower among private practices compared with other facility types, although not significantly (Table 1). Private practices were significantly more likely to accept private health insurance, and less likely to offer Ryan White services and discounted or sliding fee schedules for those who qualified, compared with other facility types. Generally, ancillary services were more likely to be available among other facility types.

**Table 1:** Descriptive statistics of major HIV care providers in the 6 county Atlanta area, overall and by practice type

	Overall		Private practice		Other facility type	
	Mean	SD	Mean	SD	Mean	SD
Available provider-hours*	99.9	97.6	89.8	84.7	117.4	117.8

	n	%	n	%	n	%
Accepted payment types						
Private health insurance^†^	36	88	26	100	10	67
Medicare	34	83	22	85	12	80
Medicaid	30	73	18	69	12	80
Discounted/sliding fee^†^	18	44	6	23	12	80
Ryan White^†^	10	24	1	4	9	60
Available ancillary services						
HIV case management^†^	12	29	2	8	10	67
Mental health services^†^	9	22	1	4	8	53
Dental care^†^	6	15	0	0	6	40
Substance abuse treatment^†^	3	7	0	0	3	20
Transportation assistance^†^	11	27	2	8	9	60
On-site pharmacy^†^	11	27	3	12	8	53
One or more services offered^†^	16	39	5	19	11	73

* HIV providers included in this count include physicians, physician’s assistants, and nurse practitioners

^†^Denotes statistically significant difference across provider type at the α = 0.05 level

#### The Engage Study

Among the 213 participants enrolled in The Engage Study, 193 (91 %) were living in the six county area, among which 163 (84 %) reported receiving HIV care from an identifiable provider located in the six county area and were included in the analysis. Of those, 100 (61 %) were black/African American, 84 (52 %) had an annual household income of less than $20,000, and 64 (39 %) did not have health insurance at the time of the survey. Median travel distance between participant residence and the last HIV care provider was 8.6 miles (IQR: 4.5, 13.4). More details about The Engage Study participants have been previously described (Dasgupta et al. 2015b).

### Discrete choice modeling

The final discrete choice model results demonstrated four spatial and provider characteristics most salient in patients selecting an HIV care provider, including travel distance to HIV care provider, available provider-hours, whether or not the clinic offered at least one ancillary service, and whether the provider offered Ryan White services. In addition, the two-way interaction between travel mode and availability of Ryan White services was retained in the final model. No other interaction terms between provider characteristics and travel mode were statistically significant.

### Development of supply access equation

Table 2 shows the parameters estimates from the final, multivariable GEE model that were applied to the supply access equation. Because availability of Ryan White services varied in importance by mode of transit, two separate parameters were included in the supply access equation reflecting these differences. The parameters from the Engage Study discrete choice model were used in the subsequent equation quantifying supply access for every ZCTA-provider pair in the study area (*a*_*ij*_) as shown below:

$$a_{ij} = \left( {\ln distance_{ij} } \right)^{ - 0.3178} \left( {ln provhrs_{ij} } \right)^{0.8797} \left( {2.2737ancserv_{ij} } \right)\left( {1.1794RW_{ij} [1 - PT_{ij} }]\right)\left( {1.9020RW_{ij} PT_{ij} } \right)$$

where i = ZCTA, j = individual providers, ln distance = natural log of the travel distance between each ZCTA centroid and provider j, ln provhrs = natural log of the number of available provider-hours at provider j, ancserv = whether provider j offers any ancillary services to patients, RW = whether or not provider j accepts patients eligible for Ryan White services. Additional information on assumptions made in developing this equation can be found in Additional File 1: Appendix S1.

**Table 2:** Parameter values used in the supply access equation for travel by public transportation and car

	Travel by car		Travel by public transit	
	Ln (odds ratio)	Odds ratio	Ln (odds ratio)	Odds ratio
Distance (natural log)	−0.3178	–	−0.3178	–
Available provider-hours (natural log)	0.8797	–	0.8797	–
At least one ancillary service available	–	2.2737	–	2.2737
Offers Ryan White services	–	1.1794	–	1.9020

### Application of supply access equation

#### Study output 1: computing population-based estimates of supply access by ZCTA

**Fig. 2:**
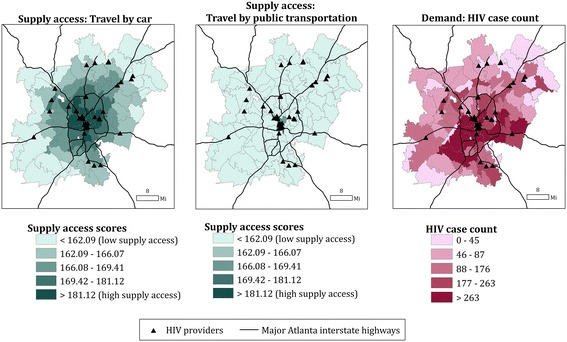
Supply access scores for travel by car (*left*) and by public transit (*middle*), and HIV case count (*right*), by ZCTA in the six county Atlanta area. The auxiliary highway, or loop route, is shown in each of the three maps and served as a boundary between urban versus suburban/rural areas

Computed supply access scores were higher in urban areas (inside the auxiliary loop route) compared with suburban and rural areas (outside the auxiliary loop route) if traveling by car (Fig. 2, left). Similar centric patterns were observed for supply access if traveling by public transportation, with lower overall scores compared with those traveling by car (Fig. 2, center). HIV cases were more concentrated in urban south, central, and east Atlanta (Fig. 2, right).

#### Study output 2: identifying underserved areas

**Fig. 3:**
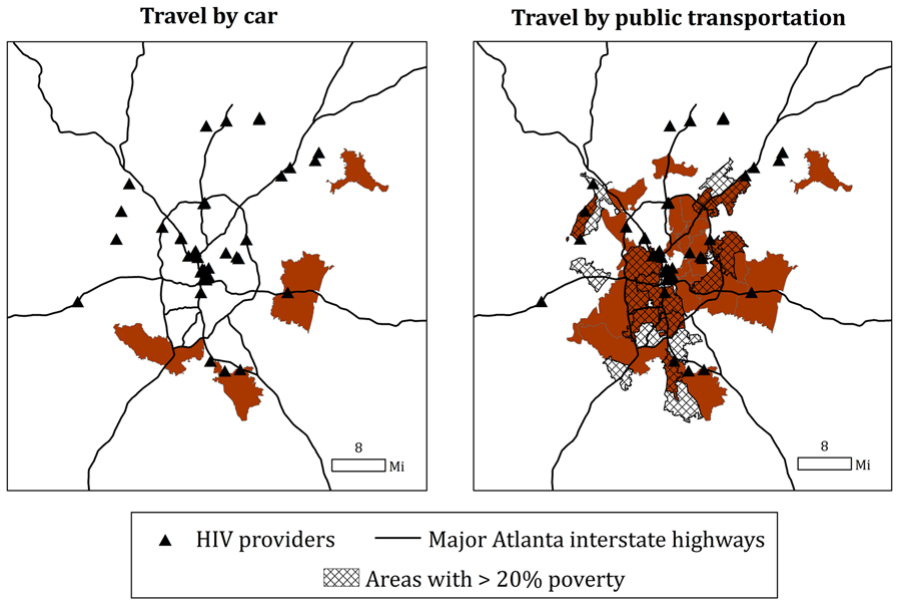
Underserved areas, defined by ZCTAs with both low supply access scores (the two lowest quintiles) and high HIV case count (the two highest quintiles), or demand, highlighted in *brown* for travel by car (*left*) and travel by public transportation (*right*). The panel on the* right* also highlights areas of high poverty (*cross*-*hatched*). The auxiliary highway, or loop route, is shown in each of the maps and served as a boundary between urban versus suburban/rural areas

For travel by car, limited areas of south and east suburban and rural Atlanta were identified as having low supply and high demand for HIV care services (Fig. 3). Only an estimated 7.7 % of HIV cases lived in these underserved areas. A majority of HIV cases in the six county area lived within five miles of the nearest HIV care provider (for additional analyses on spatial proximity to clinics, refer to Additional file 3: Figure S2). However, much of south and east Atlanta, including both urban and suburban/rural areas, was identified as underserved if traveling by public transportation; an estimated 64.3 % of HIV cases resided in these areas. Approximately 38.9 % of these underserved areas coincided with high poverty ZCTAs.

## Discussion

### Principal results

In this study, we developed and applied a novel tool to (1) quantify spatial access to HIV care services (supply) and (2) identify underserved areas, with respect to HIV case burden (demand), in the six county Atlanta area. We built on previous models of spatial access by incorporating multiple facets of spatial access, including availability, accessibility, affordability, and accommodation, into a single measure. Based on the results, travel distance to a clinic, weekly available provider-hours, availability of ancillary services, and whether Ryan White services were offered at a clinic were significant factors in choosing a provider. Supply access scores were higher in urban areas for both modes of travel, but lower overall scores were observed when traveling by public transit. Underserved areas were more pronounced when traveling by public transit in south and east Atlanta. These results corroborate what has been echoed by HIV providers locally but has not been previously quantified.

In this analysis, persons taking public transportation to attend appointments were more likely to attend a provider offering Ryan White services compared with those who traveled by car. This may be because reliance on public transportation is associated with factors related to poverty, such as dependence on Ryan White services. Access to public transportation has been shown to be positively correlated with poverty (Glaeser et al. 2008). In Atlanta, access to public transportation is greater in areas of high HIV prevalence, high poverty, and low household vehicle ownership (Dasgupta et al. 2015a, b). Expanding Ryan White funding in areas of need may improve healthcare access for persons relying on both public transit and publicly-funded medical services who might have fewer options for care. Further, expanding co-located ancillary services in clinics accepting Ryan White patients may be beneficial in improving HIV care engagement (Conviser and Pounds 2002).

Taking public transportation has been associated with greater commute times and increased number of modes of travel (Beirao and Cabral 2007; van Vugt et al. 1996), which can be an added inconvenience in accessing health services. One way to address these travel-related barriers to care might be to improve connectivity and frequency of available transit (1) between different clinic locations within a medical system that are highly accessed by HIV patients, and (2) in areas of high poverty and HIV prevalence, in which a large proportion of people living with HIV may rely on public transportation as a sole means for travel. Although rarely utilized to specifically deliver HIV care in the United States, mobile clinics may also help address travel-related barriers for those with limited transportation options. Mobile clinics have been used to administer other types of medical care successfully in the United States (Song et al. 2013; Edgerley et al. 2007).

Increasing overall HIV service availability in underserved areas would improve spatial access to HIV medical care in Atlanta. However, construction of new clinics in areas of greater need is limited by local funding. One more cost-effective way to increase service availability, particularly in rural areas with limited funding and providers, is to use traveling medical teams to provide care to different clinics throughout the week. Another method is by offering HIV medical care within structures of already existing clinics, particularly in those located in underserved areas.

The results from this study demonstrate how access to health services can have meaningful geographic variation without relying solely on spatial proximity (distance to clinic) as the reason. In particular, if access was characterized uniformly across Atlanta in this study, underserved areas for travel by public transportation, which otherwise had a sufficient supply environment if traveling by car, would not have been identified. We used a methodologically novel approach to incorporate multiple dimensions of spatial access into a single, comprehensive measure to assess the supply of services around Atlanta. We accounted for the fact that spatial access may be defined based on varying sets of characteristics for different people. We expanded on work which defined access based solely on spatial proximity to services, and highlighted potentially underserved areas in the city spatially and by mode of transportation. The analytic methods employed in this analysis were novel, but demonstrated just one of many ways to conceptualize access based on previously described frameworks and availability of data. This work should be seen as a starting point for others to improve on the modeling strategies utilized in this study so conceptualizations of spatial access to healthcare services can be further developed and refined.

Finally, it is important to consider potential interventions to improve spatial access to HIV care as the body of literature on this subject continues to grow. If the results presented can be corroborated in future studies, conducting cost-benefit analyses on implementing certain interventions, some of which are suggested in this paper, may be a key next step in addressing the mismatch between the healthcare system and HIV patients living in the Atlanta area.

### Limitations

There were some aspects of access not captured in the supply access scores, including quality of patient navigation systems for each clinic. Implementing patient navigation systems have been championed as a way to help patients steer through complexities of a healthcare system (Fischer et al. 2007; Vargas and Cunningham 2006), reduce barriers to care engagement, and improve retention in care over time (Bradford et al. 2007), and should be incorporated in future measures of spatial access. Although neighborhood contextual factors have been shown to be associated with health-seeking behaviors and outcomes (Piccolo et al. 2015; Carroll-Scott et al. 2013; Mohnen et al. 2012), we assumed that many of these factors were addressed through components of the healthcare system. For example, having flexible payment options and offering travel vouchers may be helpful for low income individuals.

The HIV care provider database may underrepresent smaller private practices treating persons with HIV infection. We did not account for whether or not HIV care providers were taking new patients, so spatial access could be overrepresented. Because the Engage Study population was only comprised of MSM, the weights in the supply access model may have been misspecified. HIV case counts used to identify underserved areas were based on ZCTA of diagnosis and not on current residence; patterns of mobility after diagnosis were therefore not captured.

Last, the supply access scores computed for travel by public transit are highly sensitive to the estimate we used to transform the scores. Future studies conceptualizing and quantifying access by travel mode should utilize more updated estimates describing the association between public transit use and HIV care attendance.

## Conclusion

Overall, spatial access to HIV care varies across the Atlanta area, and may be lower in south and east Atlanta among those using public transit. Characterizing supply environment spatially, based on discrete choice modeling, and separately by mode of transportation taken to attend HIV medical visits may be useful in bridging gaps between patient needs and service availability. If corroborated, these results could have a tremendous impact on policy surrounding allocation of resources devoted to HIV prevention and treatment. Further, this methodology can serve as a foundation for quantifying access to care in other cities with high HIV prevalence and disparate access to services, and generally, as a framework for how access to healthcare is conceptualized.

## Additional file

10.1186/s40064-016-2515-8 **Appendix S1**. Additional information on modeling assumptions used in the study.
10.1186/s40064-016-2515-8 **Figure S1**. Sensitivity analyses conducted to demonstrate the estimated range of supply access values by mode of transportation.
10.1186/s40064-016-2515-8 **Figure S2**. Additional information on spatial proximity to the nearest HIV provider.

## Abbreviations

HIV: human immunodeficiency virus
AIDS: acquired immunodeficiency syndrome
ZCTA: zip code tabulation area
GEE: generalized estimating equations
MSM: men who have sex with men
