# Supplementary material for: Development of a comprehensive measure of spatial access to HIV provider services, with application to Atlanta, Georgia
Source: Springerplus. 2016 Jul 4;5(1):984. doi: 10.1186/s40064-016-2515-8 (PMC4932000; doi:10.1186/s40064-016-2515-8)
Supplement: Supplementary file 1 — 10.1186/s40064-016-2515-8 Appendix S1. Additional information on modeling assumptions used in the study. [file 40064_2016_2515_MOESM1_ESM.pdf]

Appendix S1: Additional information on modeling assumptions used in the study

Step 1: Discrete Choice Modeling

Discrete choice modeling has been used to weight components that define access to healthcare services previously [1]. This type of modeling provides one way to understand and quantify the significance of certain characteristics in choosing one provider over other available options. Subsequently, the most significant predictors of provider choice may compositely be indicative of, or serve as a proxy for, access to care. Data from the Engage Study were used in a discrete choice model to estimate characteristics most important in selecting a provider, by mode of transportation taken to attend visits. We assumed that the participants in the Engage Study (and the choices they made) represented all people living with HIV in Atlanta.

One of the primary assumptions of discrete choice modeling is that the user's choice is made to maximize utility, based on individual circumstances and constraints [2]. We assumed the study area contained a finite set of HIV care providers, and that each HIV-positive participant chose one provider out of the set of all possible providers after comparing the utility of each option. Thus, the structure of the dataset reflected an epidemiologic matched case-control study. Each case represented a "successful" pair (a participant with the provider chosen) and controls represented all other pairs (the same participant with all providers that were not chosen), resulting in a 1:40 matched case-control dataset. No restrictions were made on the available pool of providers from which to choose based on distance or commute time. Because each participant served as his own control, there was no need to include individual-level characteristics in the model.

Step 2: Development of Supply Access Equation

Spatial gravity modeling assumes an inverse exponential relationship between travel distance and access. Following other work on spatial access to medical care, we assumed an inverse exponential relationship between patient's choice for a provider and both travel distance and available weekly provider-hours. For instance, supply access scores for facilities that were closer received a greater weight than those further away [1]. Thus, distance and provider-hours were included as the natural log of the resulting odds ratio. Other provider-related characteristics were assumed to have a linear relationship with spatial access, and the corresponding odds ratios from the discrete choice model were included as coefficients in the final supply access equations.

A generic form of a_{ij}, representing the supply access score for zip code tabulation area (ZCTA)-provider pair ij, is shown below:

$$a_{ij} = \prod_{all\ c} \alpha_{c(ij)} x_c * \prod_{all\ c} {x_c}^{\beta c(ij)}$$

where the first product sequence represents the set of characteristics for which a linear relationship with supply access was assumed, and the second product sequence represents the set of characteristics with an assumed exponential relationship with supply access. In this equation, c = each characteristic

included in the supply access equation, α = odds ratios corresponding to parameter c from the discrete choice model, β = the natural log of the odds ratio corresponding to parameter c from the discrete choice model, and x_c = value of characteristic c in the supply access equation. Forty-one a_{ij} scores were calculated for every individual ZCTA, where each of the scores corresponded to an individual HIV provider in the study area (represented by letter i).

Step 3: Application of supply access equation

For every zip code tabulation area (ZCTA), 41 supply access scores were generated for travel by public transit, and 41 supply access scores were generated for travel by car. Each of these scores represented supply access from the population-weighted centroid of ZCTA i to provider j, given the sum of individual provider-related characteristics, road distance between the centroid and provider, and the mode of transit.

Specifically, where i = an individual ZCTA and j = an individual provider, the sum of supply access scores for a single ZCTA would be:

$$A_{i\,(car)} = \sum_{all\,j} a_{ij\,(car)}$$

$$A_{i\,(PT)} = \sum_{all\,j} a_{ij\,(PT)}$$

Although the supply access equation synthesized multiple dimensions of spatial access into a single measure, it did not account for the barrier in HIV care attendance shown to be associated with taking public transit, versus a car, among people living with HIV in Atlanta [3]. For instance, although the urban core has a greater availability of public transit and a higher density of HIV services, those living in urban areas and traveling by public transit may have longer commute times, reducing overall spatial access and actual use of healthcare services.

Thus, to account for the barrier associated with taking public transit and make the scores between the two modes of travel more comparable, we fixed the total sum of supply access scores for public transportation to be a proportion of that for travel by car. Previously, we showed that when controlling for race, income, and health insurance status, rates of HIV care attendance were lower among Engage Study participants who took public transportation, compared with those who traveled by car (RR: 0.84, 95% CI: 0.70, 1.01) [3]. We transformed the sum of scores for travel by public transportation based on the point estimate from this measure of association. In other words,

$$\Big(\sum_{all\,i}\sum_{all\,j} A_{ij}\Big)_{PT} = \Big(\sum_{all\,i}\sum_{all\,j} A_{ij}\Big)_{car} * (0.84)$$

where PT = travel by public transportation and car = travel by car.

References

1. Rosero-Bixby L. Spatial access to health care in Costa Rica and its equity: a GIS-based study. Social Science and Medicine 2004; 58(7): 1271-84. doi: 10.1016/S0277-9536(03)00322-8.

2. Ben-Avika M LS. Discrete Choice Analysis: Theory and Application to Travel Demand (Transportation Studies); 1985.

3. Dasgupta S KM, Rosenberg ES, Sanchez TH, Reed L, Sullivan PS. The effect of commuting patterns on HIV care attendance among men who have sex with men (MSM) in Atlanta, Georgia. JMIR Public Health and Surveillance 2015; 1 (2): e10. doi: 10.2196/publichealth.4525.
