# Supplementary material for: Development of a comprehensive measure of spatial access to HIV provider services, with application to Atlanta, Georgia
Source: Springerplus. 2016 Jul 4;5(1):984. doi: 10.1186/s40064-016-2515-8 (PMC4932000; doi:10.1186/s40064-016-2515-8)

Figure S1: Sensitivity analyses conducted to demonstrate the estimated range of supply access values by mode of transportation

Figure S1a. Estimated range of supply access values in the six-county Atlanta area for travel by public transportation based on estimated association between public transportaton use and rate of HIV care attendance.

The supply access scores for traveling by public transportation were transformed based on the estimated relationship between use of public transportation use and realized access (RR: 0.84, 95% CI: 0.70, 1.01). The panel in the middle shows the distribution of supply access when the scores are transformed based on the point estimate (RR = 0.84) and was presented in the main body of the paper. The panels on the left and right use the lower and upper bounds, respectively, of the confidence interval of the association between public transportation use and realized access.

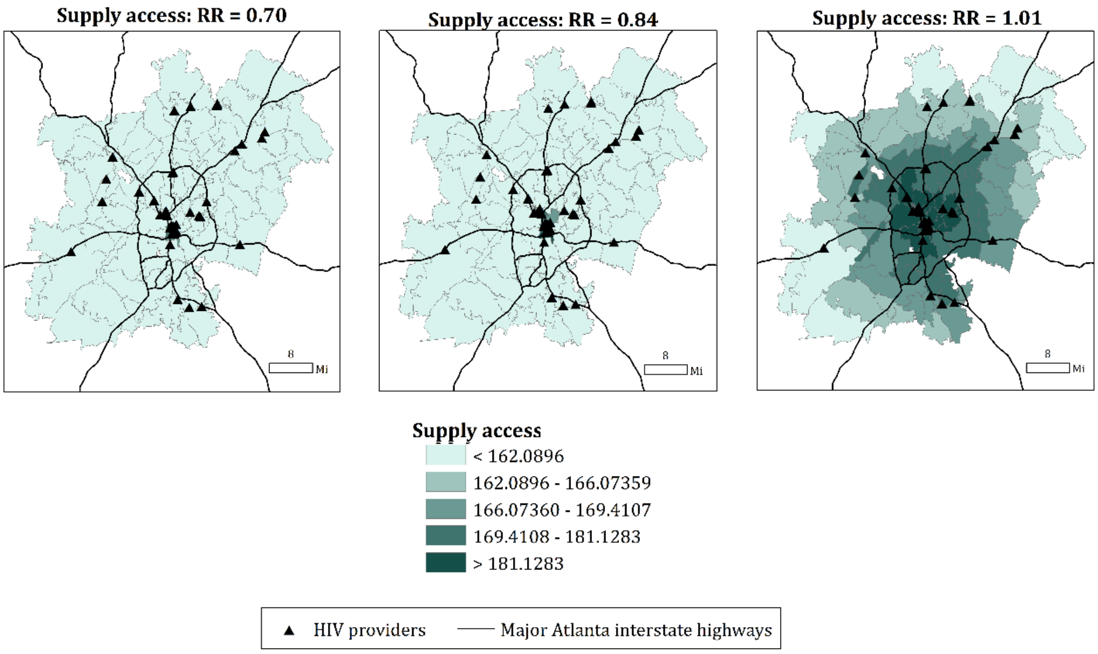

Figure S1b. Estimated range of underserved areas in the Atlanta six-county area, highlighted in maroon, for travel by public transportation, based on results from Figure S1a.

The panel in the middle shows the distribution of supply access when the scores are transformed based on the point estimate of the association between public transportation use and realized access (RR = 0.84). This panel was presented in the main body of the paper. The panels on the left and right use the lower and upper bounds, respectively, of the confidence interval of the association between public transportation use and realized access. If transforming supply scores by the point estimate, an estimated 64.3% of HIV cases are living in underserved areas, if traveling by public transportation. Based on estimations from the lower and upper bounds of the estimated association, this proportion ranges from 1.2% to 68.8%.

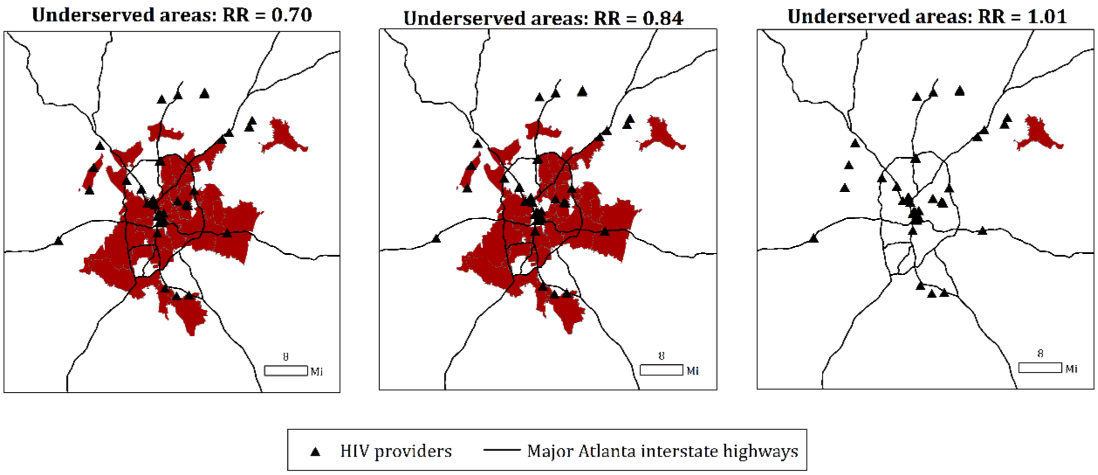

Supplement: Supplementary file 2 — 10.1186/s40064-016-2515-8 Figure S1. Sensitivity analyses conducted to demonstrate the estimated range of supply access values by mode of transportation. [file 40064_2016_2515_MOESM2_ESM.pdf]
