# Supplementary material for: Development of a comprehensive measure of spatial access to HIV provider services, with application to Atlanta, Georgia
Source: Springerplus. 2016 Jul 4;5(1):984. doi: 10.1186/s40064-016-2515-8 (PMC4932000; doi:10.1186/s40064-016-2515-8)

Figure S2: Additional information on spatial proximity to the nearest HIV provider

Using the Google maps API, we calculated driving distances and commute times between population-based centroids of ZCTAs and each Atlanta-based HIV provider. We used these computed driving distances and commute times, as well as population-based HIV case counts by zip code tabulation area (ZCTA), to estimate the cumulative percentage of HIV cases living within defined thresholds of average driving distances (miles) and commute times (minutes) to the nearest HIV provider.

Figure S2a. Cumulative proportion of all persons with diagnosed HIV infection in the six-county Atlanta area living within defined thresholds of driving distances (miles) from population-weighted ZCTAs to the nearest HIV care provider. The dotted lines highlight the median distance to the nearest HIV provider.

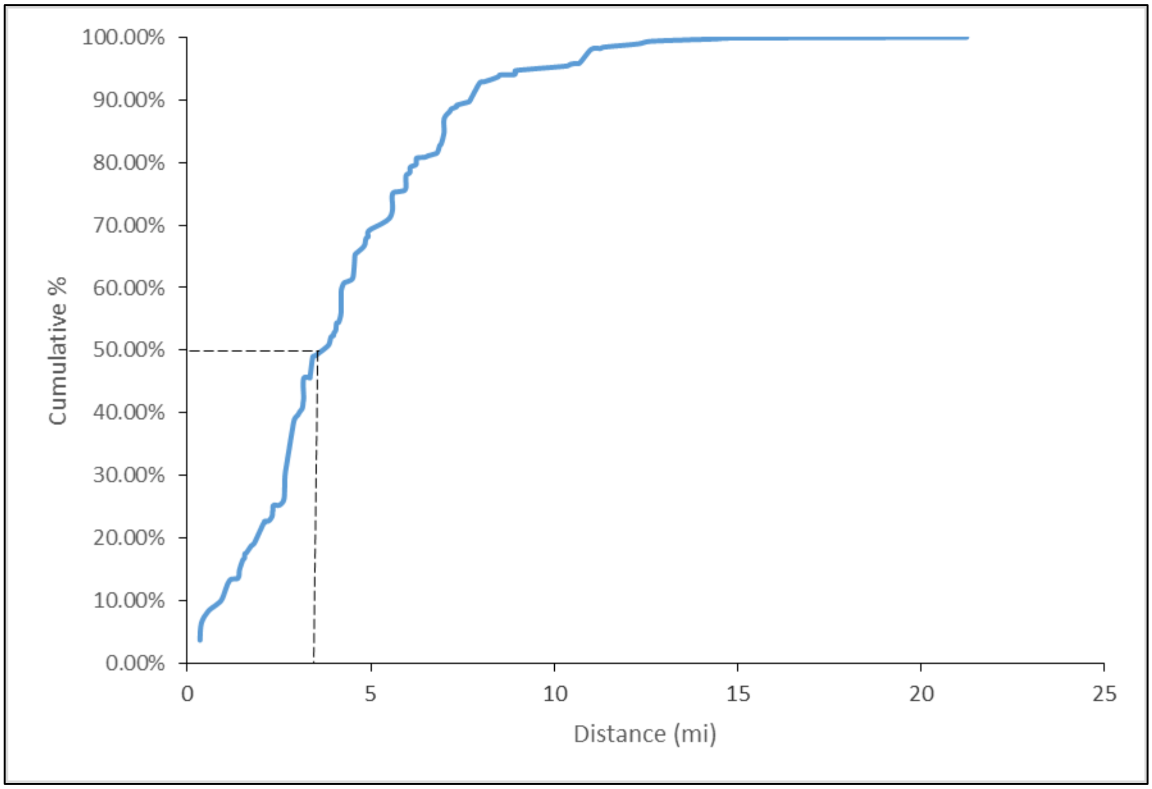

Figure S2b. Cumulative proportion of all persons with diagnosed HIV infection in six-county Atlanta area living within defined thresholds of commute time (minutes) of the nearest HIV care provider from population-weighted ZCTAs, stratified by mode of transportation. The dotted lines highlight the median commute times in minutes to the nearest HIV provider by mode of transportation.

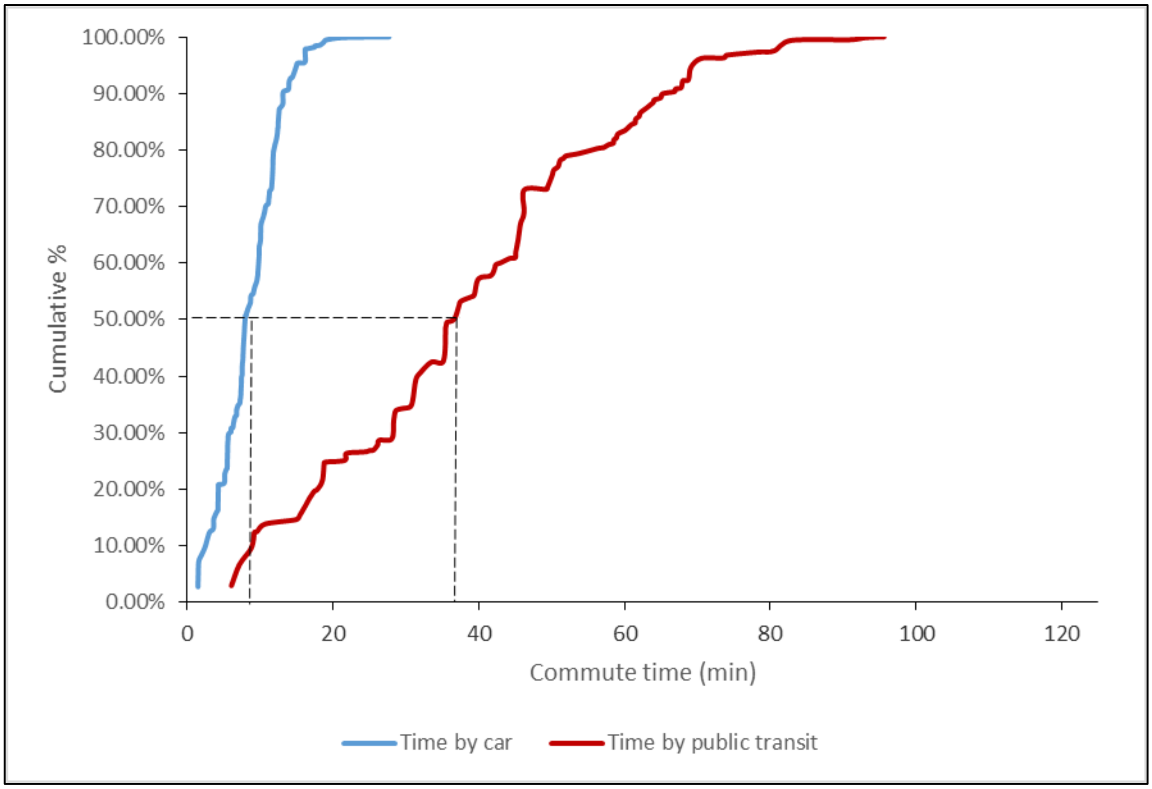

Supplement: Supplementary file 3 — 10.1186/s40064-016-2515-8 Figure S2. Additional information on spatial proximity to the nearest HIV provider. [file 40064_2016_2515_MOESM3_ESM.pdf]
